# On the feasibility of country-specific and country-general explanations for the increase over time in psychosomatic complaints among Nordic adolescents

**DOI:** 10.1177/14034948241299877

**Published:** 2024-12-02

**Authors:** Håkan Stattin, Charli Eriksson

**Affiliations:** 1 Department of Psychology, Uppsala University, Uppsala, Sweden; 2 Department of Learning, Informatics, Management and Ethics, Karolinska Institute, Stockholm, Sweden

**Keywords:** Mental health, time trends, psychosomatic complaints, sex differences, country-general and country-specific explanations

## Abstract

**Aims:**

This study examines the evidence for similar increases in psychosomatic complaints among 15-year-olds in the Nordic countries over the period 2002–2022. A distinction is made between the level and shape of these time trends.

**Methods:**

A dataset from the Health Behaviour in School-aged Children survey from 2002 to 2022 was used. Time trends for psychosomatic complaints were analysed for five Nordic countries: Denmark, Finland, Iceland, Norway and Sweden.

**Results:**

A significant increase in psychosomatic complaints over the last two decades was found among 15-year-old boys and girls in all countries, especially among girls. The shapes of the time trends were very similar for adolescents in all Nordic countries. There were significant differences in the time trends between the countries. Here, the countries with high or low levels of psychosomatic complaints in 2022 were largely the same as those with high or low levels of psychosomatic complaints years earlier.

**Conclusions:**

**The high degree of similarity observed in the shapes of the time trends for psychosomatic complaints among Nordic adolescents, as evidenced by this study, suggests that explanations for the observed increases in these psychosomatic complaints should be sought in conditions common to the five countries. However, country-specific explanations are more likely to be candidates for understanding differences in the levels of these time trends. The potential for differentiation between shapes and levels using the aggregate technique when comparing countries also provides an opportunity to empirically examine country-general and country-specific explanations for trends in measures in other areas of research.**

## Background

The monitoring of health behaviours among young people on an international scale provides crucial information to policies, programmes, and practices interested in the evolution of young people’s physical and mental health over time. Through repeated surveys, in particular the Health Behaviour in School-aged Children (HBSC) study (a collaborative cross-national project of the World Health Organization that tracks changes in a nationally representative sample of school children every 4 years), there is a monitoring system in place to enable the comparison of health trends in boys and girls in more than 50 countries. The HBSC study offers distinctive opportunities for comparative research on account of its high quality, extensive coverage, comprehensive scope, and pertinent content [1]. The study employs a public health/epidemiological approach, encompassing surveillance, the examination of populations at risk, the identification of trends, and the delineation of risk factors. The present study was primarily concerned with elucidating the question of country differences in the analysis and comprehension of trends in one of the most extensively studied mental health indicators: adolescents’ psychosomatic complaints. In the existing literature, psychosomatic complaints have been considered a particular marker of mental health problems and are intimately linked to adolescents’ stress reactions – unpredictable, uncontrollable and overloaded conditions in their everyday life [2].

Previous studies, today covering 1986 to the present, have shown a diversity of trends in psychosomatic complaints between countries. The overall conclusion is that diversity prevails [3–6]. In the 2015 study by Ottová-Jordan and colleagues [5], covering the survey years 2002 to 2010, there was a large variation in prevalence rates between countries and survey years, and there was no clear overall increasing or decreasing international trend. However, northern European countries showed an increase in psychosomatic complaints over time, which was not observed in many other regions. When the 35 HBSC countries from 2002 to 2010 were grouped according to the particular shape of their psychosomatic trends over the survey years, Ottová-Jordan and colleagues found both linear increases or decreases and U-shaped or inverted U-shaped trends [5]. Of the five countries that showed a linear increase in psychosomatic complaints, four of them, Denmark, Finland, Greenland and Norway, were Nordic countries. What followed were empirical studies of these time trends in psychosomatic complaints in the individual Nordic countries, including Sweden [7–10], Norway [11–14], Denmark [15, 16] and Finland [17, 18]. Several of these studies, which also include other surveys of regional samples in the Nordic countries [7, 19, 20], conclude that there has been a significant increase in psychosomatic complaints in recent decades for both boys and girls, with a particularly marked increase for 15-year-old girls, but a considerably lower increase for younger age groups.

These temporal trends vary according to the time periods covered by the different studies. However, few empirical studies, if any, have found other types of age and time trends in psychosomatic complaints. This remains the case in the reviews and empirical studies that have combined information on time trends from individual Nordic countries [21–23]. However, limitations of these studies include a lack of differentiation between the shapes and levels of trends across survey years and a reliance on analyses of linear trends without considering other potential trends [22, 23]. In this study, we examine changes in psychosomatic complaints among adolescents in the Nordic countries over the last 20 years. Do trends in adolescents’ psychosomatic complaints in the Nordic countries show similar patterns and levels across survey years over the last two decades? How might these trends be related to country-specific and general explanations?

The Nordic countries are geographically close, share many cultural and value similarities and can perhaps best be described as welfare states with a high standard of living, a strong emphasis on education and gender equality, full employment, a high degree of equality, and high levels of taxation and public spending on welfare [24] and public health promotion [25]. Similar time trends have been reported for other behaviours among young people in the Nordic countries. For example, smoking and alcohol consumption among adolescents in the Nordic countries have declined over the last two decades [26, 27]. This raises the question of whether psychosomatic complaints among adolescents in the Nordic countries also show a common trend over time.

How should the time trends in psychosomatic complaints for the Nordic countries of Denmark, Finland, Iceland, Norway and Sweden be explained? In principle, if the shapes of these time trends differ markedly between the Nordic countries over the years, this would suggest that country-specific explanations are needed. The social situation in one country might explain the development of psychosomatic problems among the young people in that country, but differently from other Nordic countries. On the other hand, if the shapes of the time trends are similar across the Nordic countries, then explanations are likely to be sought in conditions that are similar across the Nordic countries. It is less clear how similarities and differences between countries in the level of time trends relate to general and country-specific explanations. High similarity in both the shape and level of time trends between countries might suggest a more cultivated country-general explanation, whereas high similarity in shape but low similarity in level might indicate that a time trend showing increases is likely to be determined by country-general factors, whereas the levels of these time trends are influenced by country-specific factors over the period considered.

To date, no study has systematically examined time trends in psychosomatic complaints – shape and level simultaneously – for the Nordic countries. In the present study, we examined whether the time trends in psychosomatic complaints for boys and girls in the Nordic countries tended to have similar shapes and levels over time. We used information from the HBSC from 2002 to 2022. We recognise that similarities in shapes and levels assume measurement invariance across countries and that non-participation bias is similar. The first assumption has been tested and accepted for the survey years 1994 to 2014 [23]. The second is more difficult to verify. To the best of our knowledge, few deviations from the data collections have been reported that would lead to biased non-participation in the Nordic countries.

## Methods

### Participants

As stated, data were obtained from the international HBSC study, a cross-sectional survey conducted every 4 years to monitor the health behaviour of nationally representative samples of 11-, 13- and 15-year-old school children in Europe, North America and Israel [28]. The present study analysed nationally representative samples of 15-year-olds in the Nordic countries of Denmark, Finland, Iceland, Norway and Sweden who participated in the 2001/2002, 2005/2006, 2009/2010, 2013/2014, 2017/2018 and 2021/2022 data collections.

Participating countries follow a standardised research protocol for sampling, survey instruments and data collection. Samples were drawn using cluster sampling, with school classes or the whole school as the primary sampling unit. Iceland invited all schools in the country and therefore had a larger sample than the other countries surveyed. Ethical approval was given by the lead institutions and agencies in the participating countries. More detailed information on the methods of the HBSC study has been reported elsewhere [28]. Participation is shown in Table I.

**Table I.** Participation in different survey years in the Nordic countries (*N* = 48,576).

	2002	2006	2010	2014	2018	2022
Denmark	1350	1515	1215	1249	919	1626
Percentage girls	52.1	51.1	53.2	54	49.2	49.4
Finland	1724	1568	2062	1951	1135	983
Percentage girls	50.5	54.1	52.5	51.6	50	52.3
Iceland	-	1865	3636	3288	2210	2889
Percentage girls	-	49.6	49.3	50.1	50.9	49.2
Norway	1600	1494	1319	905	858	981
Percentage girls	51	47.2	46.7	51.8	51.6	49.1
Sweden	1198	1508	2041	2683	1621	1423
Percentage girls	50.4	50.8	49.8	51.4	52.2	48.7

*Note:* Data were not collected in Iceland in 2002.

### Measures

The main measure was the HBSC Symptom Checklist, also known as the Psychosomatic Symptoms measure. The scale is a non-clinical measure of subjective health symptoms. It consists of the core question, ‘In the last 6 months, how often have you experienced . . .?’, followed by eight items: headache, stomachache, backache, feeling low, irritability or bad temper, feeling nervous, difficulty falling asleep, and feeling dizzy. The response categories, reverse-coded, are (1) rarely or never, (2) about every month, (3) about every week, (4) more than once a week, and (5) about every day. The eight items form one factor [29, 30] and have been shown to have acceptable test–retest reliability and internal consistency [4, 12, 31]. Here, alpha reliability was 0.87 in 2022, varying between 0.84 and 0.88 across countries. Gender was coded as boy (0) or girl (1).

#### Data analysis

Polynomic trends have previously been used to describe fluctuations in psychosomatic complaints over time in different countries [5]. However, in the present case, with a direct focus on comparing the specific shapes of trends in psychosomatic complaints between the five Nordic countries, we decided to carry out the analyses at the aggregate (country) level rather than at the individual level. The data can be considered essentially free of measurement error as the samples from each country were large and the averages therefore had very small sampling errors. To determine similarities in shape, for each pair of countries we calculated the correlations between them for the six observation points. Such correlations can be tested for significance, assuming that the six time points are randomly selected from a large population of time points. Then *r* > 0.82 was required for *p* < 0.05. Before making these calculations, we standardised the means for each country by subtracting the country’s mean calculated over all six time points from the value for each time point. A measure of overall profile similarity was the median of the 10 pairwise correlations with the addition of the lowest and highest correlation.

### Results

**Figure 1.**
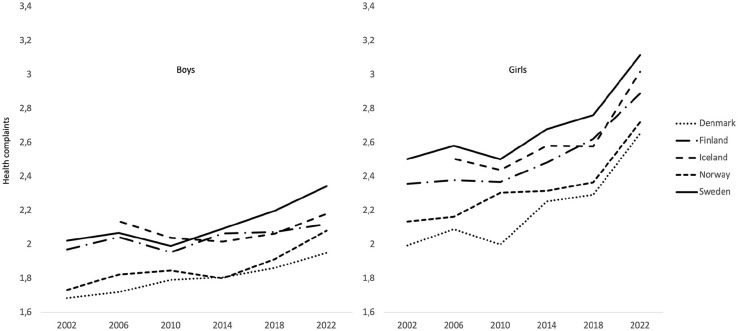
Mean of psychosomatic complaints among boys and girls across all survey years

We calculated changes in psychosomatic complaints among 15-year-olds from 2002 to 2022 for each Nordic country separately and then compared them between countries. The time trends for boys and girls are shown in Figure 1. They showed striking similarities in shape, but the profiles of the time trends for boys were different from those for girls. For boys, there was an increase from 2010 to the survey year 2018, followed by a decrease in 2022. In addition to girls having higher levels of psychosomatic complaints than boys in all survey years, the time trends for girls showed a steeper increase from survey year to survey year from 2010 to 2022. We concluded that the changes in psychosomatic symptoms between 2002 and 2022 were quite similar for adolescents in the five Nordic countries, but different for boys and girls.

**Figure 2.**
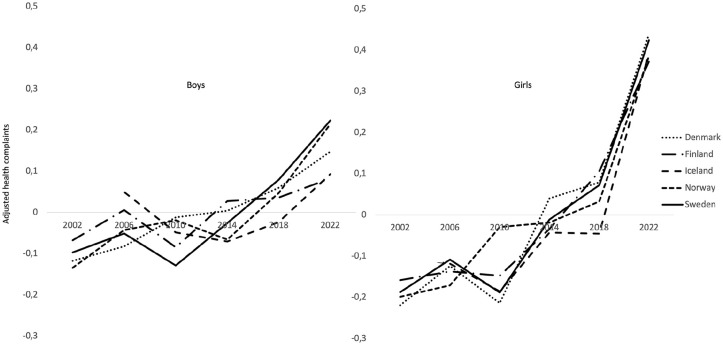
Means of psychosomatic complaints for boys and girls across all survey years, adjusted for differences in levels between countries.

To determine the *similarities in shape*, we standardized the means for each country by subtracting from each survey year the country’s mean calculated over all six survey years. These time trends, with the level differences between countries removed, are shown in Figure 2 for boys and girls separately. The results show striking profile similarities over time for both boys and girls. The shape of psychosomatic complaints over the six survey years for one country can be compared with the shapes for all other countries (resulting in ten pairwise correlations). To test these correlations for significance, a measure of overall profile similarity was calculated by taking the median of the ten pairwise correlations between the shapes of psychosomatic complaints in the five countries. This profile similarity (including the lowest and highest correlation) showed a median of 0.73 (0.36–0.93) for boys. This median is below the .82 limit which is required for *p* < 0.05. A closer examination showed that boys in Iceland reported comparatively high levels of psychosomatic complaints when they entered the HBSC survey in 2006. When this value was excluded, the profile similarity increased to 0.85 with seven of the ten correlations above .82. The results for females showed a median of 0.95 (0.94–0.99). We conclude that the time trends from 2002 to 2022 for psychosomatic complaints are very similar for boys and girls in the five Nordic countries. It is noteworthy that the changes in psychosomatic complaints during this period differ between the sexes. The increase over time is most pronounced among girls.

Following up the two Figures, the analysis of the data from the entire period spanning 2002 to 2022 indicate a slight increase in Cohen’s d for boys in Iceland and Finland. In contrast, the increase was of a medium magnitude in Denmark, Norway and Sweden (Denmark 0.41, Finland 0.20, Iceland 0.05, Norway 0.51, Sweden 0.43). The effect was of a medium to large magnitude for girls (Denmark 0.86, Finland 0.66, Iceland 0.56, Norway 0.70, Sweden 0.73). The survey year 2014 represents a breakpoint in the data, marking a point at which the increase from 2002 reached a Cohen’s d of 0.21 for boys and 0.34 for girls. The observed increase from 2014 to 2022 in all countries, as measured by Cohen’s d, was more pronounced for girls than for boys when compared to the increase observed between 2002 and 2014 (0.43 vs. 0.34 for girls). However, the latter period exhibited a somewhat lower magnitude for boys (0.18 vs. 0.21). Therefore, the majority of the observed increase in psychosomatic complaints among girls occurred between 2014 and 2022.

To examine *differences in levels* between countries, we calculated the mean value of psychosomatic complaints for each country and compared these means across the five countries in 2006 and 2022. In the last survey year, 2022, post-hoc analyses with SNK (Student-Newman–Keuls) showed significant differences between all countries (*F* (4, 7901) = 52.22, *p* < 0.001, eta^2^ = 0.03), with adolescents in Sweden having the highest level of psychosomatic complaints and those in Denmark having the lowest (significant differences: Sweden > Iceland > Finland > Norway > Denmark). Almost the same differences between countries, *F* (4, 7945) = 100.71, *p* < .001, eta^2^ = 0.05, were found in a one-way ANOVA with SNK post hoc test for the 2006 survey year (when Iceland participated in the HBSC survey for the first time): Sweden and Iceland > Finland > Norway > Denmark. In summary, the differences in psychosomatic complaints between the five Nordic countries found in 2022 already existed 16 years earlier.

#### Robustness of the findings

The measure of psychosomatic complaints used here is based on the means of the separate items for psychological and somatic symptoms. Several previous publications have instead reported the proportion of adolescents who reported experiencing at least two subjective health complaints more than once a week. We recalculated the results using this proportional measure and found almost identical shape and level results. For example, the profile similarity (including the lowest and highest correlation) showed a median of 0.85 (0.63–0.99) for boys (excluding Iceland in 2006) and a median of 0.96 (0.93–0.99) for girls.

### Discussion

In this study, we asked whether the increase in psychosomatic complaints between 2002 and 2022 would be similar for boys and girls in the five Nordic countries: Denmark, Finland, Iceland, Norway and Sweden. The answer seems to be yes. The changes in the shapes of psychosomatic complaints from 2002 to 2022 were about the same in each of these five countries. The implication of these findings is that it is unlikely that the changes in psychosomatic complaints over time are due to conditions that are unique to a particular country. What is happening in one of the Nordic countries that could explain the increase in psychosomatic complaints over time is likely to parallel conditions that also apply to the increase in psychosomatic complaints over time in the other Nordic countries. We therefore concluded that the explanations for the similarities in the shapes of the time trends from 2002 to 2022 are likely to be similar for all the Nordic countries. This hypothesis needs to be tested through empirical analyses of potential explanatory conditions within each Nordic country, that is, it remains to be identified which particular conditions are responsible for the shapes of the psychosomatic complaints in the Nordic countries over this period among 15-year-old boys and girls. These analyses could include economic, stress or social media conditions that are similarly experienced in these countries [32, 33].

It is not uncommon for increases in psychosomatic complaints in a given country to be attributed to specific conditions prevailing in that particular country. For example, in 2018 the Public Health Agency of Sweden [34] attributed the observed increase from 1984 to 2015 in psychosomatic complaints to stress experiences among adolescents, particularly school-related stress due to deficiencies in the Swedish school system, as well as characteristics of the Swedish labour market. However, there was a paucity of empirical evidence to support this assertion. Such country-specific explanations are unlikely, given the comparable temporal trends in psychosomatic complaints among adolescents in the other Nordic countries.

There were differences in the levels between the Nordic countries. But they were as evident in 2006 as they were in 2022, with Swedish adolescents scoring highest on psychosomatic complaints and Danish adolescents scoring lowest. These country differences also need to be explained. They are most likely due to country-specific conditions. Perhaps surprisingly, such conditions may be related to language. When adolescents in different countries are asked about their mental health, they respond in their own language, and the specific questions and response options may be translated somewhat differently from country to country [23].

The literature has offered many explanations for the increase in mental health problems among young people in the Nordic countries, too many to summarise here. However, these explanations have not distinguished between level and shape differences in the time trends between the countries. It is difficult to avoid the conclusion that the relevant explanatory factors for the level differences between the Nordic countries are country-specific (the level differences are about the same today as they were several years ago), but the explanatory factors for the increase in the time trends in psychosomatic complaints are more general for all five Nordic countries (the shapes are similar across the survey years).

Finally, numerous published studies that we have referred to have considered the development of mental health problems among young people over time to be synonymous with the increase in psychosocial symptoms that has occurred over the last three to four decades. However, these studies fail to acknowledge that adolescents’ perceived health status and life satisfaction remain stable over time in the Nordic countries [35]. It would be unwise to rely on a single measure in reaching conclusions about young people’s mental health.

#### Strengths and weaknesses

In this study, we employed data at the aggregate level (country level) to examine the level and shape of trends over time in psychosomatic complaints between the five Nordic countries, rather than at the individual level. We contend that these methods are intuitively clear and highlight the necessity to distinguish between the level and shape of time trends.

The high degree of similarity between countries in the shape of the time trends suggests that the explanations for the similarities from 2002 to 2022 are likely to be similar for all the Nordic countries. The significant differences in the levels of time trends between countries point to longer-term country-specific explanations. We conclude that level and shape analyses of time trends across countries for different public health indicators are likely to be more sensitive to changes in public health indicators over time than simply reporting linear, quadratic, cubic or other types of trends from analyses of linear relationships [5].

### Conclusions

Previous explanations of time trends in adolescents’ psychosomatic complaints between the Nordic countries should be treated with caution. They have not distinguished between similarities and differences in the shape and level of time trends for adolescents in the Nordic countries. Systematic analyses are required to elucidate how levels and shapes of time trends in adolescents’ health indicators other than psychosomatic complaints relate to country-specific and country-general explanations.
